# Contribution of women’s fisheries substantial, but overlooked, in Timor-Leste

**DOI:** 10.1007/s13280-020-01335-7

**Published:** 2020-05-08

**Authors:** Alexander Tilley, Ariadna Burgos, Agustinha Duarte, Joctan dos Reis Lopes, Hampus Eriksson, David Mills

**Affiliations:** 1 WorldFish Timor-Leste, Ministry of Agriculture and Fisheries, Av. Nicolao Lobato, No. 5, Comoro, Dili, Timor-Leste; 2 WorldFish, Jalan Batu Maung, Batu Maung, 11960 Bayan Lepas, Penang Malaysia; 3 Muséum National d’Histoire Naturelle, Département Homme & Environnement, Laboratoire Patrimoines locaux, Environnement et Globalisation (PALOC), French National Research Institute for Sustainable Development (IRD), ANR Popei-Coll, UMR 208 Paloc (IRD-MNHN), CP 135 - 57, rue Cuvier, 75231 Paris Cedex 05, France; 4 Australian National Centre for Ocean Resources and Security (ANCORS), University of Wollongong, Squires Way, Wollongong, NSW 2500 Australia; 5 Australian Research Council Centre of Excellence for Coral Reef Studies, James Cook University, Douglas, Australia

**Keywords:** Coastal livelihoods, Food security, Gender, Gleaning, Poverty, Women

## Abstract

A greater understanding of gendered roles in fisheries is necessary to value the often-hidden roles that women play in fisheries and households. We examine women’s contributions to household food and income using focus group discussions, market surveys, and landings data in six communities in Timor-Leste. Women were actively fishing more days per month than men. Gleaning was the most frequent activity and 100% of trips returned with catch for food and/or income. Mollusc and crab catches were common and exploitation appeared targeted on a dynamic reappraisal of changing food values and changing estimates of group needs. With as many as 80% of households in coastal areas involved in fishing, and at least 50% of women fishing, this highlights the current lack of women’s engagement as a critical gap in fisheries management approaches. The current androcentric dialogue limits social-ecological understanding of these systems and the potential for their effective stewardship.

**Electronic supplementary material:**

The online version of this article (10.1007/s13280-020-01335-7) contains supplementary material, which is available to authorized users.

## Introduction

There is growing recognition that small-scale fisheries (SSF) are an irreplaceable source of food and nutrition to millions of poor people around the world, particularly in coastal regions (Golden et al. 2016; Hicks et al. 2019; Österblom et al. 2020). Locally caught aquatic foods (i.e. finfish, seaweeds, molluscs like cockles and octopus, and crustaceans like crabs and crayfish) are an accessible yet under-recognised source of bioavailable micronutrients (Bogard et al. 2017). And, even when only conducted on a seasonal or part-time basis, these fishing activities are often a critical element in household economies (Béné et al. 2016; Mills et al. 2017). Fisheries in many countries are data blind to women’s contributions (Mills et al. 2011; Harper et al. 2020), which perpetuates relative or total exclusion from management, and has in several instances led to women’s isolation from the resource on which they depend. Awareness of women’s fisheries catch and effort is a first, but alone not an adequate step, to include women’s voices and consideration of their activities in resource management decisions (Kleiber et al. 2014). Gender equal fisheries policy that explicitly strengthens food and nutrition security, depends on our understanding of the magnitude and variability of fisheries’ contributions, and the gendered factors that affect the distribution, access, and use of fisheries resources (Fröcklin et al. 2014; Weeratunge et al. 2014; de la Torre-Castro et al. 2017). Ultimately, research that highlights the relationship between fisheries and poverty, the welfare function of fisheries and the role of fisheries in local food security are as important for policy development as fisheries production information (Allison and Mills 2018).

Open access, low-entry-cost fishing activities conducted by women that contribute small but regular input often represent a crucial food security activity for isolated communities (Hockey et al. 1988; Quinn and Davis 1997; Beitl 2011). This is particularly relevant in the small-island developing states, where reliance on coastal resources is high. In American Samoa, women and child fishers landed 32% of the annual catch from inshore environments, 68% of which came from gleaning (Hill 1978): the manual collection of food in shallow intertidal areas (Chapman 1987). Research on gleaning provides useful insight into the dynamics of poverty and resource reliance in fishing communities (Charles 2011).

Timor-Leste is a small-island developing state with at least 60% of the population being food insecure and more than 50% of children stunted due to micronutrient deficiency (Molyneux et al. 2012; von Grebmer et al. 2018). Foraging for wild food is a key coping strategy to food insecurity (da Costa et al. 2013) and access to marine resources drive large differences in per capita fish consumption between inland and coastal areas (AMSAT International 2011). Androcentrism in national fisheries management is entrenched. While currently under revision, policy, law and data collection systems have largely ignored fisheries that are not conducted from a boat, thereby excluding most women fishers. Hence, the direct and indirect roles women (and children) play in fisheries are not well understood at all. There are very few sex-disaggregated data with which to assess activities and contributions, and very few women are involved in decision-making at national and municipal levels (López-Angarita et al. 2019). In this paper, we aim to characterise women’s fisheries in the context of SSF in Timor-Leste to gain a better understanding of who fishes, where and with what, the drivers affecting fishing activities and the role that fishing and fishing-related activities play at the household level. In doing so, we attempt to highlight the unrecognised contribution that women and their fisheries catches represent across Timor-Leste, and reflect on the impact this may have on management and development priorities.

## Materials and Methods

### Focus group discussions and fisher interviews

**Fig. 1:**
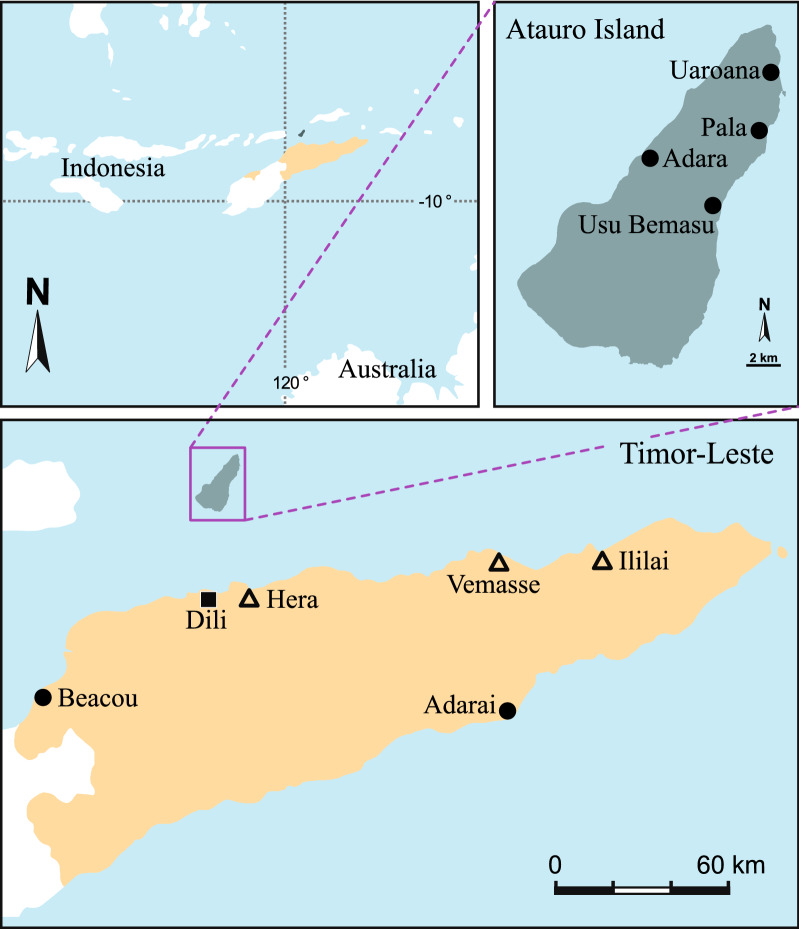
Map of community sites surveyed throughout Timor-Leste. Solid circles represent communities where focus group discussions and fishing diary data were collected. Hollow triangles represent communities where only focus group discussions were conducted

Focus group discussions (FGD) formed of 5–15 women fishers were carried out in 9 communities throughout Timor-Leste (Fig. 1) during February and March 2017 and women were interviewed individually after data collection for specific clarifications. Name, age and household size were recorded for each participant. Respondents were asked to describe seasonal patterns of marine resource use, gear types, target species, market prices and ecological trends. An inventory of species considered important for food or commercial ornamental trade by coastal communities was developed in December 2017 and August 2018 on Atauro Island by surveying gleaners catches, household shell waste dumps and conducting market surveys (*n* = 5). Local names, as well as the local ecological knowledge regarding the different species, were recorded. Taxonomic identification of species was conducted at the marine invertebrate collection of the French Museum of Natural History in Paris. Finfish and crustacean species landed were identified by trained data collectors referencing a photographic list of 130 known local species. Unknown species were listed as such and submitted along with a photograph.

### Fishing activities

Findings from interviews and focus group discussions (FGD) across all communities informed the structure of a fishing activities diary and gleaning data collection form (Appendix S1), which was distributed to FGD participants in 6 of the communities (Fig. 1). All of these women were asked to record their daily fishing activities and income for at least one continuous two-week period between March and August 2017. For each fishing activity recorded, women noted the date, the method/gear (hand line, net, spear or gleaning), if the catch was consumed by the household or sold, or both, and the total income earned from catch sales per fishing method.

Women fishers’ activity coefficient (AC) by location was estimated to compare with vessel activity coefficients from men’s fishing reported in (Tilley et al. 2019b), by calculating the proportion of days fished from the total number of days in the sampling period. This was then reported as the number of days per month fishing. Income was not normally distributed so differences in income according to location and fishing method were tested using a Wilcoxon test with Chi-squared approximation.

For gleaning activities, fishers used an additional form to record a greater level of detail on species groups that were landed, as well as the income from these groups, and trip duration. The form provided for gleaning activities was prepopulated with commonly targeted gleaning species groups identified by FGD participants. These were: fish, moray eels (separated by respondents, so this categorisation was maintained), octopus, squid, “snails” (cockles, clams, mussels, oysters, snails, winkles and abalones), crabs, shrimps and other. Income was only recorded per trip, and not per species group so it was not possible to evaluate species market price information from landings data. This information was gathered through interviews and FGDs. As a measure of relative abundance or commonness of each species (i) in the catch composition by location, an index of relative importance (IRI) (adapted from Kolding 1989) was used:

$$\% {\text{IRI}}_{i} = \frac{{\% N_{i} \% F_{i} }}{{\mathop \sum \nolimits_{j = 1}^{6} (\% N_{j} \% F_{j} )}} \times 100$$

where $\% N_{i}$ is percentage number of each species group $i$ of the total catch and $\% F_{i}$ is the percentage frequency of occurrence of each species in the total number of samples, summed over all species groups from $j = 1$ to 6.

## Results

### Focus group discussions and fisher interviews

**Fig. 2:**
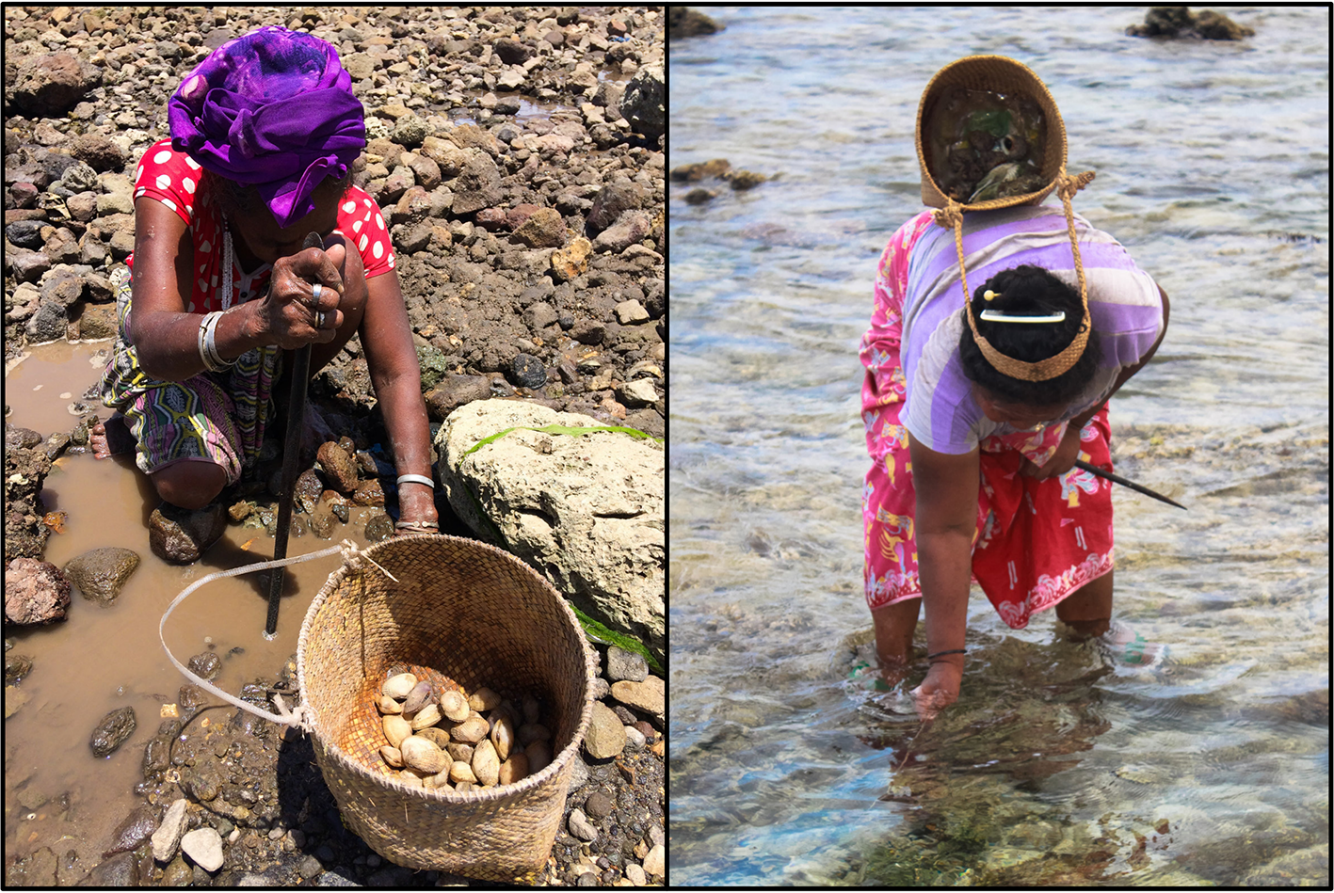
Women gleaners: Digging for cockles *Asaphis violascens* (left pane) and probing for octopus at low tide (right pane). Photos by A. Tilley

Focus group discussions indicated that women actively fished using one or more gear types in 9 out of 10 locations. Only the site of Vemasse in Baucau reported that women do not actively fish or glean. Most gleaners stated that they went gleaning every day for different purposes (food, household income, and hobby). There was variation in gleaning techniques and target species. Gleaning trips would target one species or group of species, using various combinations of gears and techniques (Table 1). Fishing classified as gleaning (*meti*) implies manual collection but may include carrying a hooked metal bar for prying up or breaking open rocks or pulling organisms from crevices (Fig. 2), a small basket for trapping (*roso*), and plant-based toxins for stunning or flushing out reef dwelling organisms. The creel carried to collect the catch is most often a basket woven from palm fronds *(bote)* (Fig. 2). Women rely on environmental signals, local knowledge and active communication to select gleaning localities and target species. Thus, keeping track of other gleaners’ activities by personal observation or information exchange is fundamental to maximise gleaning catches. While gleaning, women pay particular attention to the disturbance and distribution of rocks and reef elements, the marks from iron bars on the reef, and freshness of sand footprints. Exchange of information regarding the village’s fishing dynamics takes place on-site, while gleaning, but also at any time during the day.

**Table 1:** Main species captured by women’s nearshore fishing activities in Timor-Leste, and their uses

Group	Main species	Usage
Fish	Reef and seagrass dwelling herbivores and grazers such as rabbitfishes and spinefoots (Siganidae), wrasses (Labridae and e.g. *Novaculichthys macrolepidotus*), surgeonfishes & unicornfishes (Acanthuridae), and parrotfishes (Scaridae and e.g. *Leptoscarus vaigiensis*)	Household consumption and income. Species are sold fresh or dried. Drying is common in remote areas (due to transportation issues/costs) In Atauro, spinefoot and wrasse species are sold grilled at the weekly market (to domestic tourists) Species of all sizes are captured, with children and youth specialising in targeting small-bodied species and juvenile fish occurring in the very shallow reef, rockpools and seagrass habitats using a small handheld Hawaiian sling comprised of a stiff metal wire powered by an elastic band (*kilat ki’ik*) (Fig. 4)
Moray	Muraenidae (e.g. *Enchelynassa canina*, *Echidna nebulosa*, *Gymnothorax* spp.)	Moray eels are a low-value species that are generally only consumed by the fisher households. However, they are relatively easy to catch as they are found in tidal pools hunting for stranded fish and invertebrates
Shrimp	*Palaemon concinnus, Metapenaeus ensis, Psalidopus huxleyi, Metapenaeopsis* spp., *Metapenaeus* spp.	Most species of shrimps and prawns are sought after, either sold during market days, used to prepare shrimp powder, or eaten locally to flavour dishes. Brackish species are fished seasonally with hand nets in river mouths or with set beach seines. Shallow water and tidal shrimp are commonly targeted by small children by overturning rocks at low tide.
Crabs	Portunidae, *Scylla serrata, Portunus armatus*	Most species of small reef crabs such as blue swimmer crabs are consumed at the household level. Large crabs (mangrove mud crabs *Scylla* spp.) are sold locally. These are rare from Atauro where very little mangrove area exists
Shelled molluscs	In sampling of Atauro Island alone, 53 species of molluscs from 31 families were recorded (40 gastropods, 10 bivalves and 3 cephalopods) (Table S1)	92% of all species listed were edible, including all bivalves. The flesh of bivalves and gastropods are used for food at the household level. Only a few species are gathered to be sold fresh for consumption such as *Asaphis violascens* and *Tridacna squamosa. Cypraea tigris, Cypraecassis rufa* and *Pinctada margaritifera* are eaten at the household level and their shell is sold. Three gastropods (*Charonia tritonis, Mauritia arabica, Monetaria caputserpentis*) and one cephalopod (Nautilus pompilius) were not consumed but their empty shell is sold to tourists (e.g. ~ $25/shell for *Charonia tritonis*). *Tectus niloticus* (~ $5-12/shell) and *Turbo marmoratus* are also sought after for adornments, decoration or jewellery. 84% of bivalves and gastropods species identified were exclusively for home consumption
Octopus	*Octopus cyanea, Callistoctopus ornatus, Octopus* spp.	Octopus was caught using an iron rod to prod and probe reef crevices (Fig. 2). The price of octopus varied according to size but was consistently one of the most valuable of all species captured across all fisheries, with a mean market price of $4.55/kg. Octopus is an important source of income (prioritised for sale) and is sold sun-dried, smoked or fresh. In Adarai octopus is baked in palm leaves (saboko) for social gatherings. Small individuals are sometimes used as bait
Squid	Loliginidae (e.g. *Sepioteuthis lessoniana, Sepioteuthis* spp.)	Squid is prioritised for sale. It is caught using a hand line and sold fresh or smoked (never observed as a dried product)
Sea urchin	Echinoidea	Urchins are gathered in seagrass areas for consumption. The roe is scooped out and cooked (steamed), or the urchin is baked whole directly on the coals then cracked open to consume.
Peanut worms	Sipunculida	Sipunculid worms are dug out of sand in seagrass areas. The rough exterior is abraded off and the inner flesh is boiled, fried or dried for consumption

In most locations, gleaning activities were timed around the tidal cycle. The most popular times to glean were the early morning and evening. When the low tide occurs outside of these times, gleaning is more arduous due to high temperatures, sun exposure, and lower catchability due to finfish and invertebrates seeking deeper refuge in reef crevices. The number of gleaners at all sites was observed to visibly increase around the spring tides due to the greater surface area of reef exposed.

**Fig. 3:**
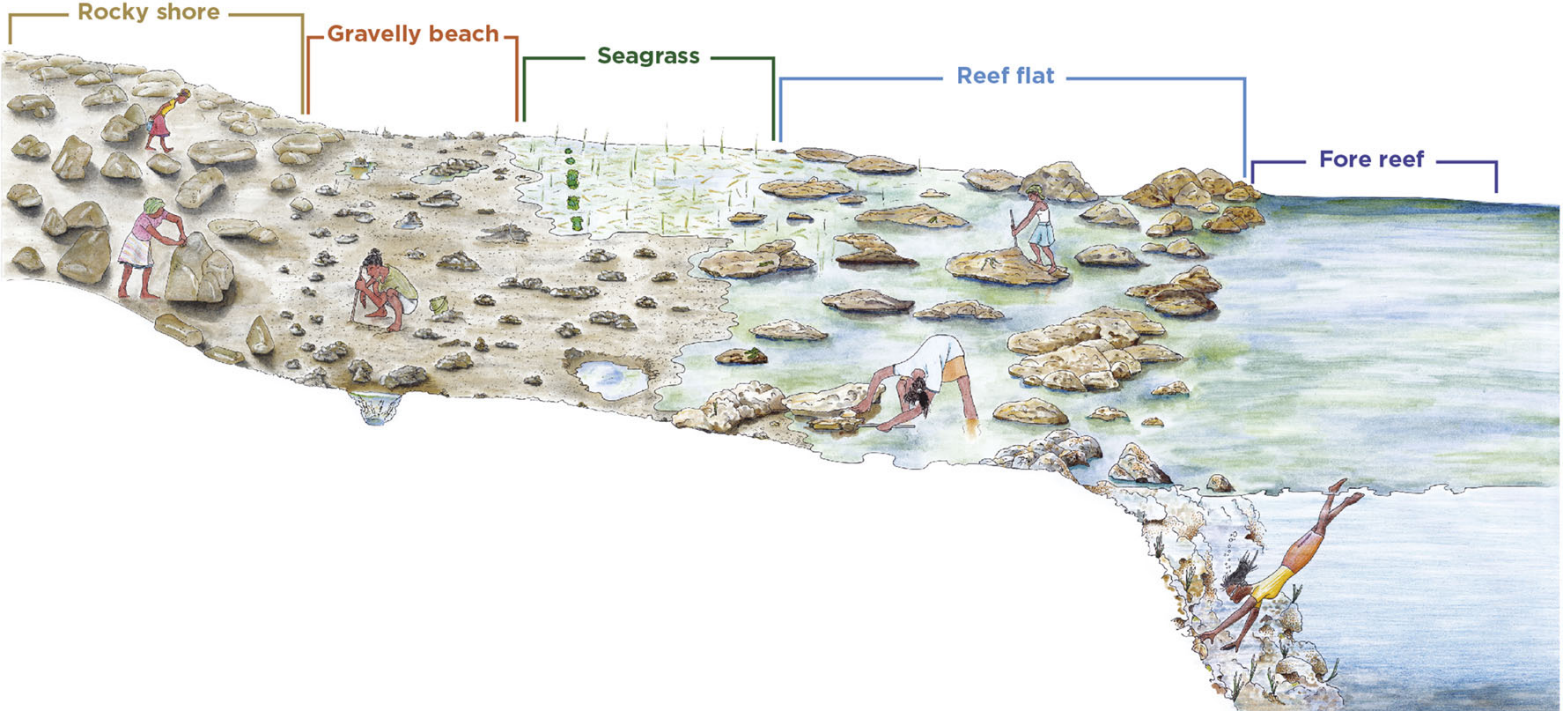
Sketch of the zonation of fishing/gleaning activities according to the ecological distribution of species. The gravelly beach is a mix of coral rubble, gravel, fine sand and coarse sands © A. Burgos and L. Billault –LRD

Interviews identified different uses according to five common intertidal environments: (i) rocky shore, where gleaners target small gastropods such as *Nerites* (snails), *Patella* (true limpets), and crab species; (ii) gravelly beach (i.e. a mix of coral rubble, gravel, fine sand and coarse sands) where an economically important bivalve, *Asaphis violascens* is found; (iii) seagrass beds, which provide habitat for finfish and molluscs while supporting the cultivation of seaweed; (iv) reef flat with the highest diversity of targeted species; and (v) forereef, where finfish, large molluscs such as giant clams, and valuable gastropods for decorative and handcraft purposes (e.g. *Charonia tritonis*, *Turbo marmoratus*, *Rochia nilotica*) are found (Fig. 3). No mangroves were present in the study villages where interviews were undertaken, so they were not identified as habitat in the exercise.

#### Species market prices

In Atauro, women in interviews reported the bivalve *A. violascens* provided the most income, along with a few small gastropods whose shells were used to make necklaces and sold to Atauro tourists. Gathering of *A. violascens* requires digging up to 20 cm in gravel substrate near the waterline (see Fig. 2, left panel)). Two hours of *A. violascens* collection typically yielded between 100 and 150 shells, and a dish containing 20–30 uncooked specimens was sold at USD $1. Assuming an average wet, shelled weight of 6 g, one sale portion would be between 120 and 180 g, indicating a sale price of between $5.50 and $8.30 per kg (Table 2). This is notably higher than the average price of fish foods from men’s landings ($2.13/kg) as reported in Tilley et al. (2019b). However, many transactions are based on ‘eyeballed’ estimations or non-standard measures such as handfuls, so accurate prices per weight are not available. The most coveted edible species at the household level were *Turbo setosus*, *Turbo chrysostomus*, *A. violascens* and giant clams *Tridacna spp.* and *Hippopus hippopus*.

**Table 2:** Prices per weight or number of individuals for consumed species groups at 6 sites across Timor-Leste, reported by women fishers in interviews and focus groups. Empty cells reflect no data available or the species group is not commonly caught or sold in the area

Species	Octopus	Fish	Shelled molluscs	Shrimp	Squid	Crab
Adara	1 kg = $6 400 g = $1.5	600 g = $1		–	–	–
Adarai	1.5 kg = $5	5 fish = $1 Morays $2–4		7–10 indiv. = $3	–	2 crabs = $3
Beacou	5 indiv. (~ 300 g) = $2	30–40 indiv. (< 5 cm) = $1		~ 30 indiv.= $1.50	2 indiv. = $2	7 indiv. = $1
Beloi	1 kg = $8 400 g = $2.5	600 g = $1	$5.50–$8.30/kg^a^	–	–	–
Biqueli	1 kg = $7 400 g = $2	600 g = $1		–	–	–
Uaroana	1 kg = $7 400 g = $2	600 g = $1		–	2 indiv. (~ 600 g) = $4	–

^a^Beloi is the focal market centre for all products being sold on Atauro Island, so the price of shelled molluscs elsewhere is likely to be substantially lower

### Fishing activities

Activity and catch data were recorded for 825 fishing trips by 32 women fishers from 6 communities between March and June 2017. Women participants ranged in age from 21 to 66 years (mean = 38.3) (Table 3). The average household size across the 6 communities was 7.0 ± 2.0 persons (compared to the national average of 5.8 (Government of Timor-Leste 2015), with the largest household size of 16 people reported in Beacou, the community with the highest mean occupancy (10.9 ± 1.8). During the sampling period in each location, the activity coefficient in days fished per month varied between 9 and 29 days, with a mean of 20 days per month across all sites (Table 3).

**Table 3:** Number of women fishers collecting data, number of trips, activity coefficient (AC) and demographic data from 6 sites in Timor-Leste

Site	*N* fishers	*N* trips	Sampling dates	AC (days/month)	Mean age (± SD)	Mean Household Size (± SD)
Adara	8	293	27/3–13/4/17	19	37.2 (± 11.6)	6.1 (± 2.2)
Adarai	3	34	11–23/5/17	28	48.0 (± 9.2)	8.7 (± 5.5)
Beacou	4	84	24/4–11/5/17	22	45.7 (± 14.6)	10.9 (± 1.8)
Beloi	5	49	22/3–11/4/17	13	45.1 (± 10.6)	6.5 (± 3.9)
Biqueli	6	125	23/3–1/4/17	29	35.9 (± 5.8)	5.3 (± 1.7)
Uaroana	6	240	15/5–24/6/17	9	34.4 (± 14.6)	7.4 (± 1.8)
Total/mean	32	825	–	20	38.3 (± 12.8)	7.1 (± 3.0)

Across all locations combined, gleaning was the most frequent fishing activity among women, representing 40% of all trips (Table 4). Different fishing methods were used on the same trip, apart from Adarai where women only conducted gleaning. Women also identified and recorded seaweed cultivation as a ‘fishing activity’, but this was excluded from the analysis of fishing activities. Women fishers did not report any fishing activities with cast nets (*dai*) or set traps (*bubur*) during the sampling period; however, small hand traps made of woven baskets (*roso*) are used in the gleaning fishery. Across all fishing activities, less than 1% of trips returned with no catch. The zero catch rate reported from men’s fisheries landings in the same communities (*N* = 2655) is 13% (Tilley et al. 2019b).

**Table 4:** Fishing activities undertaken by women in each location shown as a proportion of the total number of trips by location

	Gleaning (%)	Handline (%)	Netting (%)	Spearfishing (%)
Adara	38	26	26	10
Adarai	100	0	0	0
Beacou	52	27	17	4
Beloi	14	78	4	4
Biqueli	38	31	17	14
Uaroana	35	29	28	8
Grand total	40	30	22	8

#### Gleaning

Gleaning had a 100% catch rate: every trip returned with something to eat, sell or both. The average duration of a gleaning trip was 3.0 ± 0.5 hours. 71% of gleaning trips provided household food only, 14% provided income only (mean = $2.16 (3.92), and 14% provided both income and food.

**Fig. 4:**
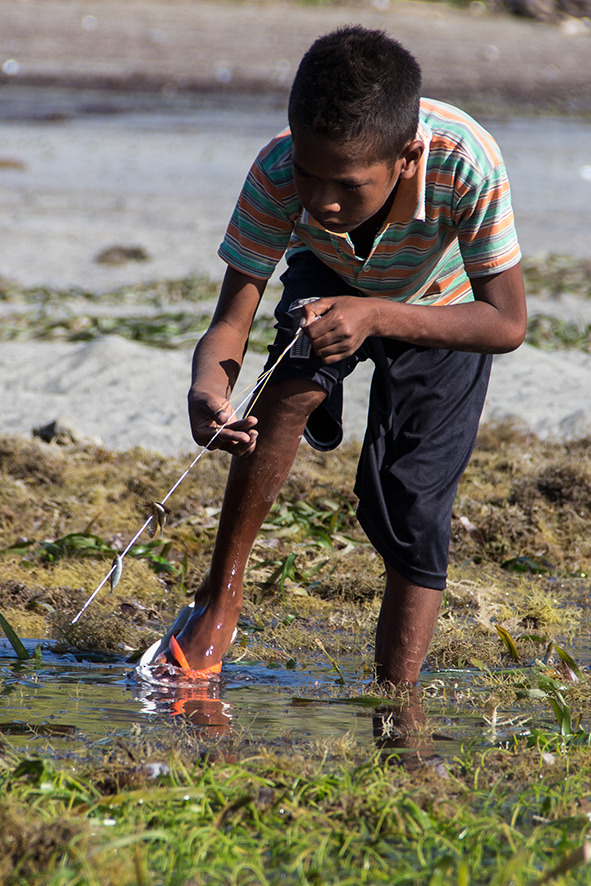
A Timorese boy fishing in a seagrass flat with a small hawaiian sling (*kilat ki’ik*). Photo by Holly Holmes

Shelled molluscs (bivalves and gastropods) were the most numerous species group in gleaning catches at all sites except Beacou, where fish dominated. Fish were the most important (IRI) species group across all sites except Uaroana (Table 5), where squid had the highest IRI. Molluscs were considerably more abundant in women’s catches from the eastern side of Atauro Island (Biqueli, Uaroana) and Adarai (where only gleaning was conducted), than other sites. Squid was only present in catches from two sites, being the most important group from Uaroana (25% IRI) and more rarely landed in Beacou (11% IRI). Octopus was relatively rare in catches from all sites, representing 17% of individuals caught in Beacou and 21% in Adarai. These two sites, perhaps correspondingly were the sites with the highest mean income from gleaning. The restricted distribution of squid catches to only two sites is likely to be due to the method by which they are caught (jigging) generally requiring access to a boat, which may not be desirable or available to women (Fig. 4).

**Table 5:** Index of relative importance of species groups landed in women fishers’ gleaning trips in Timor-Leste. Proportional importance sums per site

	Crab (%)	Moray (%)	Shells (%)	Fish (%)	Octopus (%)	Shrimp (%)	Squid (%)
Adara	19	5	29	43	4	0	0
Adarai	4	2	16	34	21	3	0
Beacou	5	2	8	44	17	15	11
Beloi	11	1	23	46	4	16	0
Biqueli	5	3	27	41	13	11	0
Uaroana	1	1	20	23	9	20	25

#### Income

**Fig. 5:**
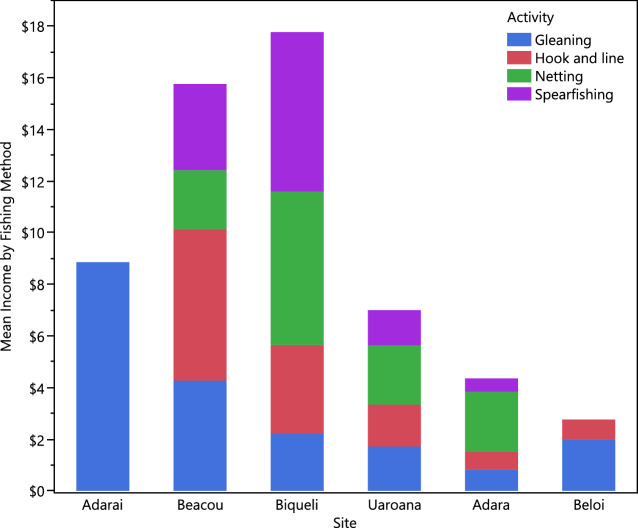
Mean income of women fishers in six coastal communities of Timor-Leste, stacked by fishing method.

The highest-earning fishing activities were gill netting and handlining, showing similar income levels (USD $ 3.31 ± 2.69 (*N* = 180) and $ 3.21 ± 1.58 (*N* = 245) per trip, respectively) (Table 6). The difference in income by location was significant (*χ*^2^ = 5, *P* < 0.0001), and by fishing activity (Wilcoxon, *χ*^2^ = 8.3328, 3, *P* = 0.0396). The highest earnings per trip across all activities seen from Adarai gleaners (Table 6). Given the isolation of Adarai on Timor-Leste’s south coast (Fig. 1), this is likely to reflect high volumes rather than high market prices. Cumulative income from all activities is more indicative of incomes from a variety of fishing methods (Fig. 5). Mean (± SD) women fisher income per trip across all activities and locations was 2.21 (± 3.91).

**Table 6:** Income rates of women’s fisheries activities represented by mean income in USD $ per trip by location and gear type

Site/gear	Gleaning	Handline	Netting	Spearfishing	Mean
Adara	0.81	0.72	2.30	0.52	1.25
Adarai	8.86	–	–	–	8.86
Beacou	4.27	5.87	2.29	3.33	4.39
Beloi	2.00	0.76	–	–	0.76
Biqueli	2.21	3.44	5.95	6.17	4.06
Uaroana	1.72	1.62	2.29	1.37	1.84
Mean	2.16	1.90	2.71	2.23	2.22
SD	3.92	3.81	3.91	4.18	3.91
Median	1	1	$0	0	–

## Discussion

In Timor-Leste, women are commonly the largest group of resource users in the intertidal reef and seagrass areas, as has been observed elsewhere in the Pacific (Chapman 1987; Harper et al. 2013; Lambeth et al. 2014). The importance of women’s fishing activities to income varied by location, but focus group participants indicated that some form of fishing was conducted by men and women in ~ 80% of households in the community. Consistent with findings from Tuvalu (Lambeth et al. 2014), many women reflected on their gleaning activity to be a social activity unto itself, or a hobby. An opportunity to spend time and talk with family and friends while being active. However, respondents agreed across all sites about the importance of gleaning during periods of low crop and horticulture production. August was mentioned as a time when gleaning was particularly plentiful and productive, corroborating findings of Mills et al. (2017) that seasonal winds during this period limit favourable fishing days and account for a dip in (‘men’s’) SSF landings.

It is often observed that fishing serves as a buffer to natural hazards and livelihood shocks for the poor (Béné 2003; Eriksson et al. 2017), providing gathered foods from open-access systems to fill the subsistence “gap” until conditions improve (Luomala 1980; Chapman 1987). Increases in gleaning and collecting are tied closely to economic crises (Gillett 2009), where low cost, non-vessel fishing methods with a high value-added ratio can bring much needed relative revenues (Gillett 2009). However, despite the short sampling period of detailed activity diaries and gleaning catch, women participants in our study were frequent and consistent users of the marine environment. Catch volumes are lower on average than landings from men’s fisheries, but the ~ 99% success rate of women’s fishing trips, compared to men’s trip catch rate of 87% (Tilley et al. 2019b), is substantial. Furthermore, our activity coefficient data suggest that women fish more regularly than men, with ~ 20 days fishing per month compared to a mean activity level of ~ 15 days per month for men (Tilley et al. 2019b).

The 71% of landings reported as solely for consumption appear to support the subsistence narrative of gathered foods for food security. Interestingly, only 14% of landings were reported as being for both food and income, which might suggest that some fishers glean more determinedly than others, rather than returning with whatever they catch and then attempting to sell it. However, these results are insufficient to make conclusions. Mollusc and crab species represent a nutritious element of Timorese subsistence diets, but only a small number of species are targeted for commercial purposes. 84% of bivalves and gastropods species were exclusively for home consumption and played an important role to secure household protein, particularly in times of shortage and bad weather. They are available throughout the year and their distribution is relatively predictable (Thomas 2007), so decisions regarding their harvest are not necessarily determined by availability, but rather rely upon continuous tallying and assessment of all potential food resources; the dynamic reappraisal of changing food values and ease of procurement; as well as the changing estimates of group needs (Waselkov 1987). Crabs and shelled molluscs play an important role in food security, and gleaners are the primary agents in monitoring day to day social–ecological and intertidal environmental dynamics. Thus, women have substantial ecological knowledge that could provide updated and essential information in the assessment of coastal change and socio-ecological vulnerability (Burgos 2016).

Recent national census results suggest that < 5% of households in Timor-Leste are involved in fishing (General Directorate of Statistics, Timor-Leste 2018), but responses to this question refer to formally recognised fishing sector activities such as handline and gill netting from a vessel, and would likely not have accounted for gleaning or other women’s fishing activities. In contrast, FGD results suggest that most households (> 80%) in a given coastal community conduct gleaning at one time or other indicating that national catch figures could be vastly under representative. Approximately 40% of the population lives in coastal areas in Timor-Leste. At ~ 560 000 people with an average household size of 5.3, this would imply that as many as ~ 84 500 households could be accessing and utilising marine resources to varying frequency. As such, the estimated consumption rates of fish for coastal areas (17.6 kg/capita/year) (AMSAT International 2011) are likely to be significantly underestimated, as well as the figure of national fisheries production. Timor-Leste is not alone. Even in the Pacific islands where gleaning fisheries are visibly important and documented in greater detail, surveys do not effectively capture the landings or consumption from these activities (Gillett 2016).

The impacts of gleaning on reef systems can be substantial and directly contribute to habitat degradation and overfishing (Andréfouët et al. 2013). Currently, fishing pressure is somewhat controlled by poor roads and limited access to ice and refrigeration (López-Angarita et al. 2019). Nearshore areas close to community sites are often chosen for fisheries closures due to ease of enforcement, but interventions rarely assess local legitimacy and transparency with all user groups, such as women fishers (Pomeroy et al. 2015). In countries such as Timor-Leste, where poverty and malnutrition statistics are some of the highest in the world (von Grebmer et al. 2018), this is likely to have serious consequences for poor and marginalised sectors of society that depend on low-entry-cost fisheries near to their homes. With increasing dependence on reef resources, comes increasing vulnerability of those involved to slide into social–ecological traps (Cole et al. 2018). Co-management processes that involve those affected by management in making management decisions (Berkes 2009) are paramount, and initial examples building on local laws and ritual practice (tara bandu) have shown some success (Tilley et al. 2019a).

Fishers across social groups and communities are not equally vulnerable to management regulations (Tilley et al. 2018), so incorporation of gendered perspectives, priorities, and aspirations is fundamental in achieving equitable, decentralised, co-managed fisheries in Timor-Leste. Addressing power and gender inequities requires the proactive development of women’s capacity and confidence to effectively participate in decision-making processes (Mutimukuru-Maravanyika et al. 2017). If investments are made in women’s education and capacity according to their aspirations, direct household nutritional gains (Miller et al. 2017) and resource sustainability improvements (Tindall and Holvoet 2008) are feasible. If however, their contributions continue to be invisible and decisions made without their participation, conservation and management initiatives could have disastrous and cascading impacts on household nutrition (Williams 2002; Bennett 2005). The impacts of management and conservation interventions on women’s fishing areas have not been thoroughly assessed, but it is clear that empowering women to make decisions is crucial to enable them to protect their fishing grounds (e.g. gleaning areas) for food security (Kleiber et al. 2018), and can have rapid and lasting benefits (Aswani and Weiant 2004; Kleiber et al. 2014).

Our characterisation and preliminary quantification of women’s fishing activities highlight the need for gender-integrated instruments in national fisheries monitoring and management. When appropriately quantified, women’s fisheries are likely to represent a significant proportion of the SSF production in Timor-Leste. There are clear power asymmetries present at different levels of governance. This study represents the first step towards equitable inclusion of women in marine management and contributes to current initiatives that are building momentum for greater gender equity in small-scale fisheries (FAO 2015, 2017).

## Electronic supplementary material

Below is the link to the electronic supplementary material.

Supplementary material 1 (PDF 929 kb)
