# Supplementary material for: Contribution of women’s fisheries substantial, but overlooked, in Timor-Leste
Source: Ambio. 2020 May 8;50(1):113–24. doi: 10.1007/s13280-020-01335-7 (PMC7708585; doi:10.1007/s13280-020-01335-7)
Supplement: Supplementary file 1 — Supplementary material 1 (PDF 929 kb) [file 13280_2020_1335_MOESM1_ESM.pdf]

Ambio

Electronic Supplementary Material
This supplementary material has not been peer reviewed.

Title: **Contribution of women's fisheries substantial, but overlooked, in Timor-Leste**

Authors: Alexander Tilley, Ariadna Burgos, Agustinha Duarte, Joctan dos Reis Lopes, Hampus Eriksson, David Mills

Appendix S1. Women's fishing activities data form – Timor-Leste

Lokalidade/Location______________________________

Naran Peskador (Fisher name)_______________

Tinan (age)_____________

Status iha uma kain (position in household)______________________

Total ema hela uma laran (N in household)________________________

Data/date														
Aktividade	Segunda		Tersa		Quarta		Quinta		Sesta		Sabdu		Domingo	
	$	Han/food	$	Han	$	Han	$	Han	$	Han	$	Han	$	Han
Ezemplu/example	$2		$5	✓		✓		✓	$3	✓				✓
Kail/hand line														
Luku/spearfishing														
Tau redi/gill net														
Budutasi/seaweed														
Meti/gleaning														
Seluktan/other														

Konteudu Meti Gleaning form	Segunda Mon	Tersa Tues	Quarta Weds	Quinta Thurs	Sesta Fri	Sabadu Sat	Domingo Sun
Ezemplu	2	1			4	5	8
Boek/*shrimp*							
Kadiuk/*crab*							
Tuna/*moray*							
Gurita/*octopus*							
Siput/*snail*							
Ikan/*fish*							
Suntu/*squid*							
(seluktan)/*other*							
(seluktan)/*other*							
Hasai foto? (✓) Photo taken?							
Oras/Hours (duration)							

Table S1. A list of mollusc species identified in the gleaning fishery of Timor-Leste

#	Species	Class	Family	Edible?	Ornamental?
1	*Anadara antiquata*	Bivalve	Arcidae	Yes	No
2	*Asaphis violascens*	Bivalve	Psammobiidae	Yes	No
3	*Barbatia sp.*	Bivalve	Arcidae	Yes	No
4	*Hippopus hippopus*	Bivalve	Cardiidae	Yes	No
5	*Mactra maculata*	Bivalve	Mactridae	Yes	No
6	*Pinctada margaritifera*	Bivalve	Pteriidae	Yes	Yes
7	*Spondylus sp.*	Bivalve	Spondylidae	Yes	No
8	*Tridacna cf. crocea*	Bivalve	Cardiidae	Yes	Yes
9	*Tridacna maxima*	Bivalve	Cardiidae	Yes	Yes
10	*Tridacna squamosa*	Bivalve	Cardiidae	Yes	Yes
11	*Angaria cf delphinus*	Gastropod	Angariidae	Yes	No
12	*Charonia tritonis*	Gastropod	Ranellidae	No	Yes
13	*Chicoreus ramosus*	Gastropod	Muricidae	Yes	No
14	*Conomurex luhuanus*	Gastropod	Strombidae	Yes	No
15	*Conus capitaneus*	Gastropod	Conidae	Yes	No
16	*Conus marmoreus*	Gastropod	Conidae	Yes	No

17	*Conus litteratus*	Gastropod	Conidae	Yes	No
18	*Conus striatus*	Gastropod	Conidae	Yes	No
19	*Conus sp.*	Gastropod	Conidae	Yes	No
20	*Cymbiola sp.*	Gastropod	Volutidae	Yes	No
21	*Cypraea tigris*	Gastropod	Cypraeidae	Yes	Yes
22	*Cypraecassis rufa*	Gastropod	Cassidae	Yes	Yes
23	*Drupa morum*	Gastropod	Muricidae	Yes	No
24	*Haliotis cf varia*	Gastropod	Haliotidae	Yes	No
25	*Harpago chiragra*	Gastropod	Strombidae	Yes	No
26	*Lambis lambis*	Gastropod	Strombidae	Yes	No
27	*Lambis millepeda*	Gastropod	Strombidae	Yes	No
28	*Latirolagena smaragdulus*	Gastropod	Fasciolariidae	Yes	No
29	*Latirus sp.*	Gastropod	Fasciolariidae	Yes	No
30	*Lentigo lentiginosus*	Gastropod	Strombidae	Yes	No
31	*Lunella cinerea*	Gastropod	Turbinidae	Yes	No
32	*Mauritia arabica*	Gastropod	Cypraeidae	No	Yes
33	*Monetaria caputserpentis*	Gastropod	Cypraeidae	No	Yes
34	*Nerita exuvia*	Gastropod	Neritidae	Yes	No
35	*Nerita polita*	Gastropod	Neritidae	Yes	No

36	*Nerita sp.*	Gastropod	Neritidae	Yes	No
37	*Oxymeris maculata*	Gastropod	Terebridae	No	Yes
38	*Patelloida saccharina*	Gastropod	Lottiidae	Yes	No
39	*Siphonaria cf. laciniosa*	Gastropod	Siphonariidae	Yes	No
40	*Rochia nilotica*	Gastropod	Tegulidae	Yes	No
41	*Tectus cf. pyramis*	Gastropod	Trochidae	Yes	No
42	*Telescopium telescopium*	Gastropod	Potamididae	Yes	No
43	*Terebralia sulcata*	Gastropod	Potamididae	Yes	No
44	*Tonna cf. canaliculata*	Gastropod	Tonnidae	Yes	No
45	*Trochus cf. maculatus*	Gastropod	Trochidae	Yes	No
46	*Turbo chrysostomus*	Gastropod	Turbinidae	Yes	No
47	*Turbo marmoratus*	Gastropod	Turbinidae	Yes	No
48	*Turbo setosus*	Gastropod	Turbinidae	Yes	No
49	*Tutufa bubo*	Gastropod	Bursidae	Yes	No
50	*Vasum turbinellus*	Gastropod	Turbinellidae	Yes	No
51	*Nautilus pompilius*	Cephalopod	Nautilidae	No	Yes
52	*Octopus*	Cephalopod	Unknown	Yes	No
53	*Squid*	Cephalopod	Unknown	Yes	No
